# N-terminal transactivation function, AF-1, of estrogen receptor alpha controls obesity through enhancement of energy expenditure

**DOI:** 10.1016/j.molmet.2018.09.006

**Published:** 2018-09-20

**Authors:** Yukitomo Arao, Katherine J. Hamilton, Sydney L. Lierz, Kenneth S. Korach

**Affiliations:** Reproductive and Developmental Biology Laboratory, National Institute of Environmental Health Sciences/NIH, Research Triangle Park, NC, 27709, USA

**Keywords:** Tamoxifen, Subcutaneous fat, Visceral fat, Obesity, Energy expenditure, Estrogen receptor alpha, Domain functionality, ER, estrogen receptor, knock-out, KO, ovariectomized, OVX, wild type, WT, high fat diet, HFD, regular diet, RD, estradiol, E2, tamoxifen, Tam, fulvestrant/ICI182780, ICI, resting metabolic rate, RMR, basal metabolic rate, BMR, glucose tolerance test, GTT, insulin tolerance test, ITT, dual-energy X-ray absorptiometry, DEXA

## Abstract

**Objective:**

Studies using the estrogen receptor alpha (ERα) knock-out (αERKO) mice have demonstrated that ERα plays a crucial role in various estrogen-mediated metabolic regulations. ERα is a ligand dependent transcription regulator and its activity is regulated by estrogenic compounds. ERα consists of two transcriptional activation domains, AF-1 and AF-2. The activities of these domains are regulated through different mechanisms; however, the specific physiological role in metabolic regulation by these domains is still unclear.

**Methods:**

We utilized an ERα AF-2 mutant knock-in mouse (AF2ERKI) to evaluate the physiological functionality of ERα transactivation domains. Due to the estrogen insensitive AF-2 mutation, the phenotypes of AF2ERKI mice are seemingly identical to the global αERKO including obesity in the females. Distinct from the αERKO, the AF-1 function of AF2ERKI mice can be activated by tamoxifen (Tam). Ovariectomized (OVX) AF2ERKI and WT females were treated with Tam and fed a high-fat diet (HFD) for 10 weeks. Additionally, indirect calorimetric analysis was performed using metabolic chambers with food intake and locomotor activity recorded for Tam-treated AF2ERKI and αERKO females.

**Results:**

Obesity in HFD-fed AF2ERKI females was prevented by Tam treatment; particularly, inguinal fat accumulation was strongly blocked by Tam treatment. Alterations in fat metabolism genes, however, were not found in either inguinal fat nor visceral fat to be Tam-regulated, even though fat accumulation was strongly reduced by Tam treatment. Indirect calorimetric analysis revealed that without alteration of food intake and locomotor activity Tam treatment increased energy expenditure in AF2ERKI but not αERKO females.

**Conclusions:**

These results suggest that the activation of ERα AF-1 prevents fat accumulation. The prevention of obesity through AF-1 is mediated by induction of energy expenditure rather than ERα AF-1 functionality of lipid metabolism gene regulation in fat tissues.

## Highlights

• AF-2 disrupted ERα mutant (AF2ERKI) females are obese with a pre-diabetic condition.
• Activation of ERα AF-1 prevented AF2ERKI obesity.
• Inguinal fat accumulation altered more than visceral fat in AF2ERKI females.
• AF-1 activation improved energy expenditure without changing activity and feeding.

## 1 Introduction

Menopausal women develop various symptoms, including weight gain and reduction in metabolism, due to the natural reduction of ovarian estrogen production. In some cases, pre- and post-menopausal women have taken hormone replacement therapy, commonly estrogen treatment, to relieve symptoms.

Animal studies using the estrogen receptor (ER) α knock-out (αERKO) mice have demonstrated that the effects and phenotypes of αERKO females are similar to those experienced by post-menopausal women [1]. The αERKO females present with obesity, impaired glucose tolerance and insulin resistance, suggesting that ERα plays a crucial role in estrogen-mediated metabolic regulation [2]. ERα is a ligand dependent transcription regulator, and its activity is modulated by estrogenic compounds. Estrogenic compounds bind to the ligand binding domain and control the functionality of the transcriptional activation domains of ERα, named AF-1 and AF-2. Cell based *in vitro* studies suggested that these activities are regulated by different mechanisms. However, the physiological role of AF-1 and AF-2 is still not known precisely. The αERKO mouse model, which does not express ERα protein, cannot define such precise domain functionality *in vivo*. For this purpose, AF-1 or AF-2 deleted mutant mice (ERaAF-1°/ERaAF-1^−/−^ or ERaAF-2°, respectively) have been reported [3]. Genetic modification in the ERaAF-1° mouse forces translation of a N-terminal truncated ERα, lacking the AF-1 domain [4]. ERaAF-2° mouse has a mutated *Esr1* genome, which deletes the corresponding sequence of the ERα AF-2 core domain, disrupting AF-2 activity [5]. The metabolic profile of ERaAF-1° mice was identical to WT; estradiol (E2) administration prevented high fat diet (HFD)-induced metabolic disturbances in ovariectomized (OVX) ERaAF-1° females [3]. In contrast, ERaAF-2° females present with obesity, impaired glucose tolerance, and insulin resistance, which is similar to the global αERKO females. Additionally, HFD-induced metabolic disturbances in OVX ERaAF-2° mice were not protected by E2 administration [3]. A previous report concluded that the AF-1 is dispensable and AF-2 is crucial for E2 dependent ERα mediated metabolic regulation [3]. The activities of AF-1 and AF-2 can be controlled selectively by various estrogenic compounds. Namely, agonist compounds, such as E2 activate AF-2 and AF-1. In contrast, selective estrogen receptor modulators (SERMs), such as tamoxifen (Tam) can activate AF-1 without AF-2 activation in a tissue specific manner [6], [7]. Additionally, SERMs compete endogenous E2 mediated ERα AF-2 activity but not AF-1, which results in the partial agonist/antagonist function of SERMs [6]. Recently, Guillaume et al. reported that the HFD induced obesity of OVX WT mice was prevented by Tam-treatment. Additionally, the effect of Tam was not observed in OVX ERaAF-1° mice [8]. Thus, the report concluded that the selective AF-1 activation is sufficient for prevention of HFD induced obesity. These observations suggest that the regulation of ERα AF-1 can be a target for controlling obesity.

Guillaume et al. also suggested that food intake reduction in Tam treated OVX WT females caused the prevention of body weight gain [8]. The importance of the connection between hypothalamic ERα functionality in behavior and metabolic regulation has recently emerged with studies of neuronal ERα KO mouse models [9], [10]. The ERα KO in the SF-1 neuron reduced energy expenditure and induced fat accumulation; POMC neuron specific ERα KO females increased food intake and body weight gain [11]. Thus, understanding of the precise functionality of AF-1 and AF-2 in ERα in metabolic regulation may lead to generation of precise therapeutic interventions for controlling ERα function appropriately in pre- and post-menopausal women.

We have reported another ERα AF-2 mutant knock-in mouse model, named AF2ERKI. The AF2ERKI mouse has mutations, which creates two amino acid substitutions on the AF-2 core domain of the ERα protein (mERα-L543A,L544A) [11]. Due to the E2-insensitive mutation of AF-2, the phenotypes of the AF2ERKI are identical to the global αERKO mice [11], [12]. In contrast to the αERKO, AF-1 specific physiological responses can be identified and rescued by ERα antagonist chemicals, such as Tam and fulvestrant/ICI182780 (ICI) in the AF2ERKI mouse [12]. We report herein that AF2ERKI females have disrupted metabolic phenotypes similar to αERKO and ERaAF-2° mice. Furthermore, HFD-induced obesity was prevented by Tam treatment to the OVX AF2ERKI females, suggesting that ERα AF-1 can control metabolic regulation, which is consistent with a previous report using a different mouse model [8]. Additionally, we performed indirect calorimetric analysis using the metabolic chambers including food intake and activity monitoring. The locomotor activity of AF2ERKI and αERKO females was significantly lower than WT, but food intake was not different between the genotypes. We found that the resting metabolic rate (RMR) of AF2ERKI and αERKO females was lower than WT females. That lower RMR of AF2ERKI was improved by Tam treatment independent from their locomotor activity and food intake. We concluded that this effect was ERα AF-1 dependent since it was not observed in αERKO females.

We suggest from our study that the specific activation of ERα AF-1 can enhance energy expenditure without changing feeding and locomotion behavior. This functionality may be a potential target of novel medications for hormone replacement therapy.

## 2 Materials and methods

### 2.1 Animals

All experiments involving animals were carried out according to US Public Health Service guidelines. Studies were approved by the National Institute of Environmental Health Sciences Institutional Animal Care and Use Committee. The generation of AF2ERKI mice has been described previously [11]. The generation of mice lacking ESR1 expression (Ex3αERKO) has been reported [13]. Heterozygote mutant mice were bred for generation of wild type (WT) and mutant homozygote mice for both lines. WT and homozygote mutant littermates were used for all experiments. Animals were maintained on a regular chow (NIH-31, Harlan Laboratory). Animals were allowed *ad libitum* access to food. The body weight was checked every week.

### 2.2 Glucose tolerance test and insulin tolerance test

Mice were fasted overnight (16 h) and injected with 2 g/kg d-glucose intraperitoneally (IP) for glucose tolerance test (GTT). Fasted mice received IP injections of 0.75 units/kg Humulin R (Eli Lilly) for insulin tolerance tests (ITT). Tail vein blood was taken to measure the blood glucose levels prior to injection and after injection at 20, 40, 60, 120, and 180 min. Nova Max glucometer (Nova Biomedical) was used for analysis.

### 2.3 Dual-energy X-ray absorptiometry (DEXA)

DEXA measurement and data analysis were performed using Lunar PIXImus (GE Medical Systems). The system was calibrated according to manufacturer's instructions prior to the data collection. The animals were anesthetized and laid on the chest during measurement. The skull was placed in its approximate position and subtracted from the data analysis.

### 2.4 High fat diet study

7–9 weeks-old mice were separated to two groups; a group of mice were ovariectomized (OVX) and the other group of mice were left intact. The mice were fed regular chow (NIH-31). Two weeks after surgery, OVX mice were implanted with a tamoxifen (Tam; 0.028 mg/day/mouse), estradiol (E2; 0.006 mg/day/mouse) or placebo pellet (Innovative Research of America). When the mice were 9–11 weeks-old, the diet was changed to high fat diet (HFD) and maintained for 10 weeks. Animals were allowed *ad libitum* access to food during this period. HFD contains 60 kcal% fat (#12492, Research Diets). GTT and DEXA were performed 2 weeks before sacrifice (on the 8th week HFD feeding). ITT was performed 1 week before sacrifice (on the 9th week HFD feeding). The body weight was recorded every week.

### 2.5 Sample collection

The animals were fasted for 4 h before euthanasia with carbon dioxide. Tissues were weighed and a part of tissue was fixed in 10% neutral buffered formalin for histological assessment, the remaining tissue was frozen in liquid nitrogen and stored in −70 °C for RNA extraction. The fat pads were collected from inguinal adipose tissue, mesenteric adipose tissue for visceral fat and interscapular brown adipose tissue. The liver tissue was collected from the left lateral lobe of liver.

### 2.6 RNA quantification

The frozen tissues were pulverized then homogenized with TRIzol reagent (Invitrogen) for extracting RNA. The DNase I-treated total RNA was reverse-transcribed by Superscript II (Invitrogen) to generate the cDNA following the manufacturer's instruction. Quantitative PCR was performed using the Fast SYBR Green Master Mix (Applied Biosystems) with cDNA and the gene specific forward and reverse primers. The sequence of primers is listed in Table 1. The Ct value was calculated by a mathematical model to quantify the relative mRNA level [14].

**Table 1:** Primers for quantitative PCR.

Gene	Forward (5′- -3′)	Reverse (5′- -3′)
*Lpl*	TCAACAAGGTCAGAGCCAAGAGAAGCAG	GCCATCCTCAGTCCCAGAAAAGTGAATC
*Dgat1*	TCTTTGTTCAGCTCAGACAGTGGTTTCA	ACCAGGATGCCATACTTGATAAGGTTCTCT
*Lipe*	CTGACTTCCTGCAAGAGTATGTCACG	GGTTCTGTATGATGCGTTCAAATTCAGC
*Pnpla2*	GAGAGAACGTCATCATATCCCACTTTAG	CTGTGATGGTATTCTTCAGCTCATAAAGTG
*Acly*	CTTCCTCAAGAACTTTCTCATTGAACCC	CGTAGAAATTGAATAGACCAGAGATGAAGC
*Αcaca*	ACACTTTCTGATTTGGGGATCTCTGGCTTAC	GCGATAAGAACCTTCTCAATTACTTTATTTCCCCC
*Fasn*	CACAGATGATGACAGGAGATGGA	TCGGAGTGAGGCTGGGTTGATA
*Scd1*	CATCATTCTCATGGTCCTGCT	CCCAGTCGTACACGTCATT
*Elovl6*	ATGAACAAGCGAGCCAAGTTTGAAC	TGACAGGTCCATTGTAAAAACTCTGGTC
*Hmgcr*	GTACATTTACTTCCAGTTCCAGAACCTAC	GAGCTATATTTTCCCTTACTTCATCCTGTG
*18s*	GTGCAGCCCCGGACATCTAA	GGAATTGACGGAAGGGCACC
*Rpl7*	AGCTGGCCTTTGTCATCAGAA	GACGAAGGAGCTGCAGAACCT

### 2.7 Histology

The formalin-fixed tissues were rinsed with ethanol then embedded in paraffin. The tissue sections were stained with hematoxylin and eosin.

### 2.8 Indirect calorimetric analysis

The study was performed separately for AF2ERKI and αERKO groups. In this study, we used 11–14 week-old intact mice which have similar body weight and lean mass between WT and mutant to exclude the parameter of body composition difference for assessing the heat production level (kcal/h/kg). The animals were maintained with regular chow (NIH-31). The mice were implanted with Tam (0.024 mg/day/mouse) or placebo pellet (Innovative Research of America) before using analysis. After 14 days treatment, the mice were single-housed in the metabolic chamber for 3 days (72 h) with regular chow to record the levels of food intake, locomotor activity, oxygen consumption and carbon dioxide production using the TSE LabMaster System (TSE). Animals were allowed *ad libitum* access to food during experiment. To allow for an acclimation period, the data from the final 48 h were used for analysis. Body weight was used for the calculation of heat production level (kcal/h/kg). Locomotor activity represented ambulatory beam breaks of movement detector.

### 2.9 Statistical analysis

All data are represented as mean ± S.E.M.. Statistical analyses were performed by GraphPad Prism 7 (GraphPad Software, Inc.). ANOVA with Tukey's multiple comparison test was performed to test for significant difference. Significance level was set at p < 0.05 for every analysis.

## 3 Results

### 3.1 Inactivation of ERα AF-2 leads to obesity and pre-diabetic condition

**Figure 1:**
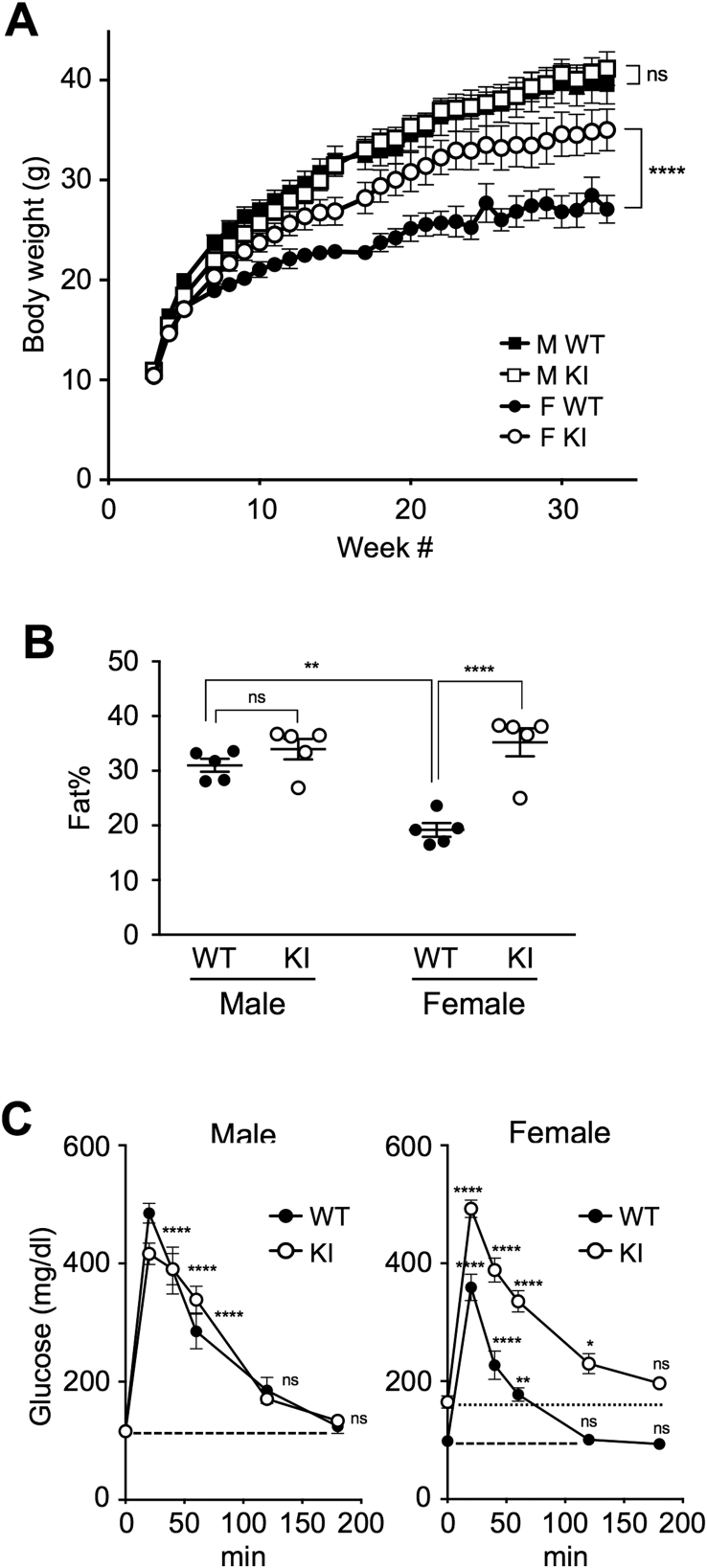
**Inactivation of ERα AF-2 leads to obesity and pre-diabetic condition in females.** (**A**) Body weight in WT and AF2ERKI (KI) male (M; square symbols; WT *n* = 7, KI *n* = 7) and female (F; circle symbols; WT *n* = 7, KI *n* = 7) mice fed regular diet (RD). The body weight is represented as mean ± S.E.M.. Two-way ANOVA was performed to indicate significant difference between genotype. (**B**) Body fat percentage of 6-month-old RD-fed WT and AF2ERKI (KI) males (WT *n* = 5, KI *n* = 5) and females (WT *n* = 5, KI *n* = 5). The value of mean ± S.E.M. is also indicated. Two-way ANOVA was performed to indicate significant difference between genotype and sex. (**C**) Glucose tolerance tests in 6-month-old RD-fed WT and AF2ERKI (KI) male (left; WT *n* = 5, KI *n* = 5) and female (right; WT *n* = 5, KI *n* = 5). The blood glucose level is represented as mean ± S.E.M.. Dotted lines show initial glucose levels of fasting animals. Two-way ANOVA was performed to indicate significant difference against initial glucose level. *p < 0.05, **p < 0.01, ****p < 0.0001; ns, non-significant difference.

Body weight of regular diet (RD)-fed AF2ERKI and wild type (WT) male and female mice was measured for 30 weeks after weaning (3 week-old). The AF2ERKI females had higher body weights than WT littermates (Figure 1A, circle symbols). In contrast, the body weights of males did not differ between genotypes (Figure 1A, square symbols). Body composition of 6 month-old female and male mice was analyzed by Dual-energy X-ray absorptiometry (DEXA). Body fat content of AF2ERKI females was significantly higher than age matched WT females and that level was similar to males (Figure 1B). When we performed glucose tolerance tests (GTT), 6 month-old RD-fed AF2ERKI females had higher serum glucose levels than WT females after fasting (164.8 ± 11.3 mg/dl and 98.4 ± 10.5 mg/dl respectively, p = 0.015). As shown in Figure 1C, the injected glucose was cleared to the initial fasting glucose level by 120 min post-injection in WT females. However, the glucose level in AF2ERKI females did not return to the initial level and was delayed until 180 min post-injection. The GTT profile of AF2ERKI males was identical to WT males (Figure 1C). These metabolic disorders (obesity and impaired glucose tolerance) observed in AF2ERKI females were strikingly similar to global ERα knock-out (αERKO) females [15], suggesting that the proper functionality of ERα AF-2 is necessary for metabolic regulation in female mice.

### 3.2 Differential fat deposition in the fat tissues of high fat diet fed AF2ERKI females

**Figure 2:**
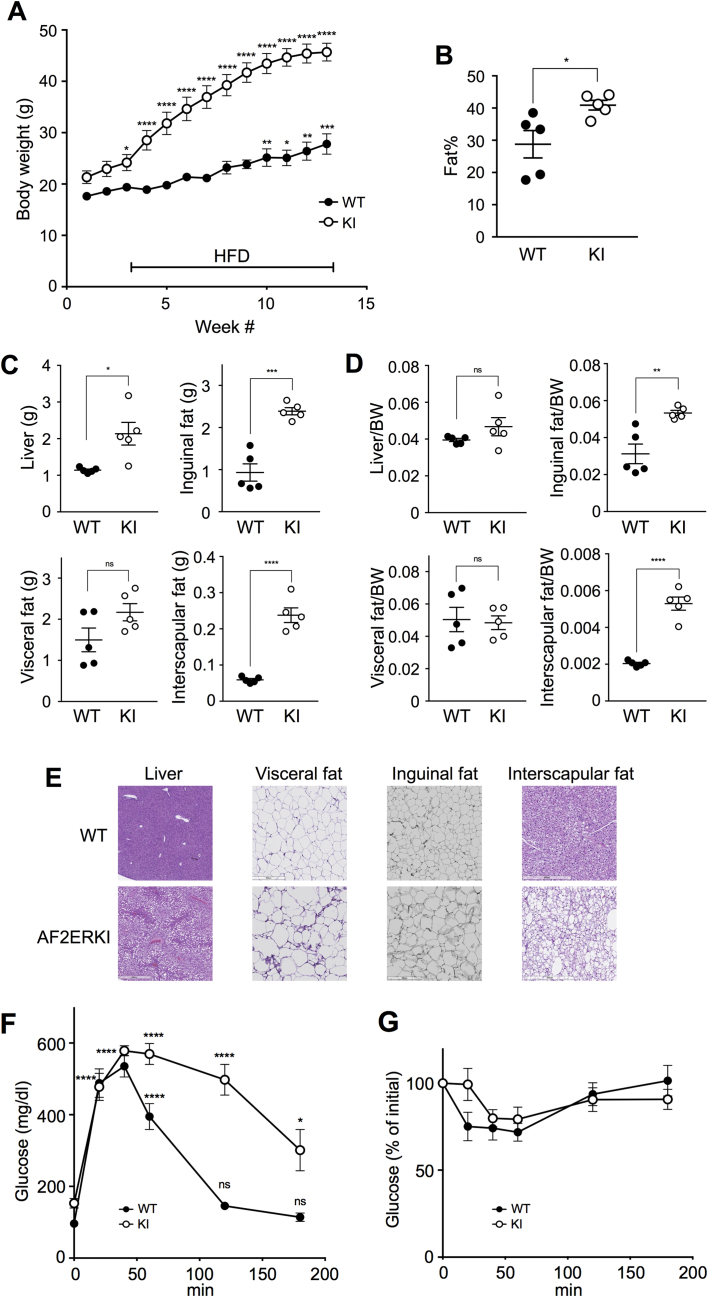
**Differential fat deposition in the fat tissues of high fat diet fed AF2ERKI females.** (**A**) Body weight in WT and AF2ERKI (KI) female (WT *n* = 5, KI *n* = 5) mice fed high fat diet (HFD) for 10 weeks. The line indicates the period of HFD feeding. The body weight is represented as mean ± S.E.M.. Two-way ANOVA was performed to indicate significant difference against initial body weight. (**B**) Body fat percentage of HFD-fed WT and AF2ERKI (KI) females. The value of mean ± S.E.M. is also indicated. (**C**) Total tissue weight (liver, inguinal fat, visceral fat, and interscapular fat) of HFD-fed WT and AF2ERKI (KI) females. The value of mean ± S.E.M. is also indicated. (**D**) Proportion of tissue weight in HFD-fed WT and AF2ERKI (KI) females. The value of mean ± S.E.M. is also indicated. Unpaired t test was performed to indicate significant difference between genotype (*B*, *C* and *D*). (**E**) Representative tissue sections of liver, inguinal fat, visceral fat, and interscapular fat from HFD-fed WT and AF2ERKI females. (**F**) Glucose tolerance tests (GTT) in HFD fed WT and AF2ERKI (KI) females. (**G**) Insulin tolerance tests (ITT) in HFD fed WT and AF2ERKI (KI) females. The blood glucose level is represented as mean ± S.E.M. Two-way ANOVA was performed to indicate significant difference against initial glucose level. *p < 0.05, **p < 0.01, ***p < 0.001, ****p < 0.0001; ns, non-significant difference.

To evaluate the effect of excess fat in the diet, AF2ERKI and littermate WT females were treated with a high fat diet (HFD) for 10 weeks. The body weight of HFD-fed AF2ERKI females was increased dramatically 1 week after HFD feeding (Figure 2A). In contrast, the body weight of WT females was increased gradually and significant gain was observed after 7 weeks of HFD feeding. As detected by DEXA, body fat content of HFD-fed AF2ERKI females was higher than that of WT (Figure 2B). The weight of fat tissues (visceral, inguinal and interscapular fats) and liver was analyzed in the 10 week HFD-fed mice. The total tissue weight of liver, inguinal fat, and interscapular fat were significantly higher in the HFD-fed AF2ERKI females compared to the WT but there was no difference in visceral fat (Figure 2C). Increased fat accumulation compared to the WT was also observed histologically in the HFD-fed AF2ERKI female liver, inguinal fat and interscapular fat but not visceral fat, correlating with tissue weight (Figure 2E). Hepatic steatosis was observed in AF2ERKI females; however, the proportion of liver weight to body weight (relative tissue weight) was the same between AF2ERKI and WT females (Figure 2D). These results suggested that ERα AF-2 mutation increased body fat accumulation; in particular, the deposition of subcutaneous fat (inguinal and interscapular fat) was significantly higher in HFD-fed AF2ERKI females compared to WT. The GTT profile showed that HFD-fed AF2ERKI females have impaired glucose tolerance (Figure 2F). Additionally, insulin tolerance tests (ITT) indicated that the insulin treatment response tended to be slower in AF2ERKI than WT females (Figure 2G).

### 3.3 Activation of ERα AF-1 prevents AF2ERKI obesity

**Figure 3:**
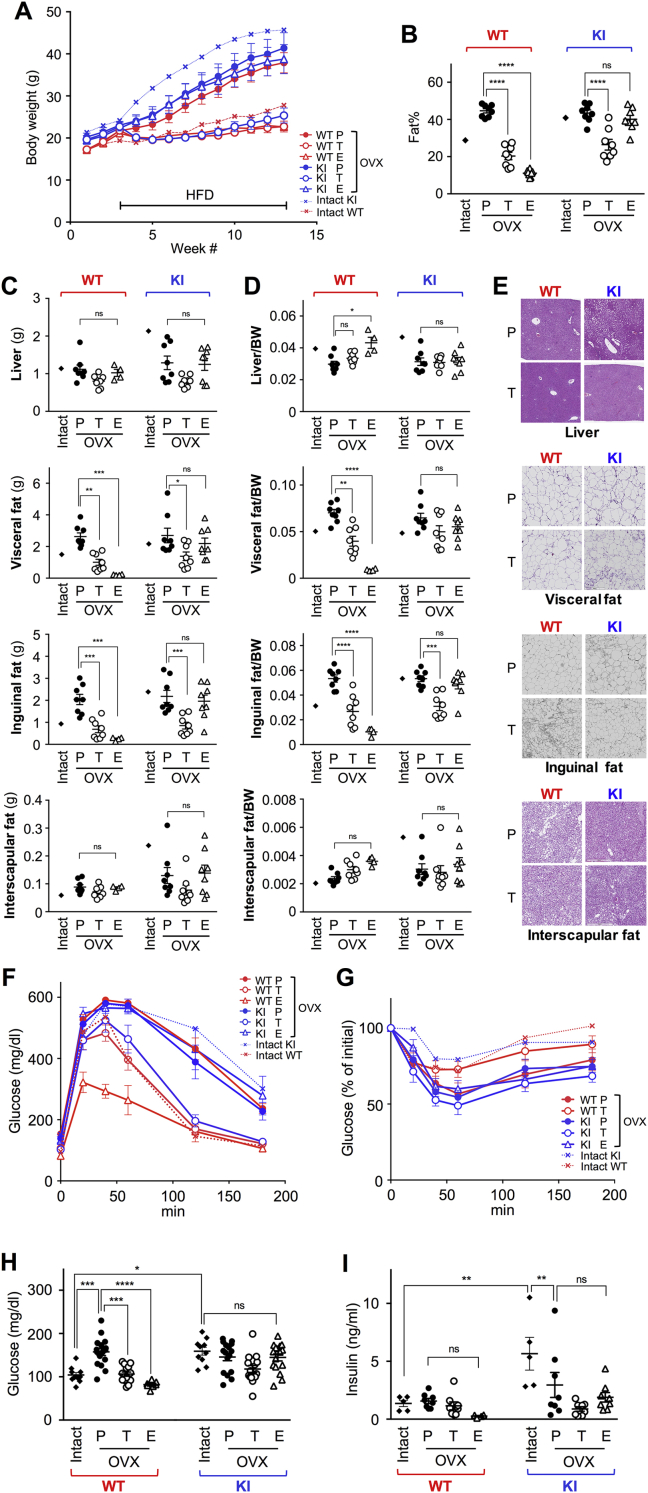
**Activation of ERα AF-1 prevents AF2ERKI obesity.** (**A**) Body weight in HFD-fed ovariectomized (OVX) WT and AF2ERKI (KI) females which implanted with placebo (P; WT n = 8, KI n = 8), tamoxifen (T; WT n = 8, KI n = 8) or estradiol (E; WT n = 8, KI n = 8) pellet for 10 weeks. The line indicates the period of HFD feeding. The body weight is represented as mean ± S.E.M.. Dotted lines indicate the body weight in HFD-fed intact WT and AF2ERKI females which are cited from Figure 2. (**B**) Body fat percentage of placebo (P), tamoxifen (T), or estradiol (E) treated OVX WT and AF2ERKI (KI) females fed HFD for 10 weeks. The value of mean ± S.E.M. is also indicated. (**C**) Total tissue weight (liver, visceral fat, inguinal fat and interscapular fat) of placebo (P), tamoxifen (T) or estradiol (E) treated OVX WT and AF2ERKI (KI) females fed HFD for 10 weeks. The value of mean ± S.E.M. is also indicated. (**D**) Proportion of tissue weight in placebo (P), tamoxifen (T), or estradiol (E) treated OVX WT and AF2ERKI (KI) females fed HFD for 10 weeks. The value of mean ± S.E.M. is also indicated. Two-way ANOVA was performed to indicate significant difference between treatment in each genotype (*B*, *C* and *D*). Average values of HFD-fed intact WT and AF2ERKI females cited from Figure 2 are shown in *B*, *C* and *D*. (**E**) Representative tissue sections of liver, visceral fat, inguinal fat, and interscapular fat from placebo (P) or tamoxifen (T) treated OVX WT and AF2ERKI (KI) females fed HFD for 10 weeks. (**F**) GTT in HFD fed OVX WT and AF2ERKI (KI) females which implanted with placebo (P), tamoxifen (T), or estradiol (E) pellet for 8 weeks. Dotted lines indicate the GTT response in HFD-fed intact WT and AF2ERKI females which are cited from Figure 2. (**G**) ITT in HFD fed OVX WT and AF2ERKI (KI) females which implanted with placebo (P), tamoxifen (T), or estradiol (E) pellet for 9 weeks. Data represented as mean ± S.E.M.. Dotted lines indicate the ITT response in HFD-fed intact WT and AF2ERKI females which are cited from Figure 2. ITT was not performed for E2-treated OVX WT females, because the mice were hypoglycemic. (**H**) Blood glucose levels in 16 h fasting animals which used for GTT and ITT. (**I**) Serum insulin levels in 4 h fasting animals. Two-way ANOVA was performed to indicate significant difference between treatments (*E* and *F*). *p < 0.05, **p < 0.01, ***p < 0.001, ****p < 0.0001; ns, non-significant difference.

We have reported that the transcriptional activity of AF-1 can be activated by tamoxifen (Tam) treatment without AF-2 activation in the AF2ERKI mouse [11]. To exclude the effect of ovarian hormones, we used ovariectomized (OVX) AF2ERKI and WT females. The OVX mice, which were implanted with a Tam, estradiol (E2) or placebo pellet, were maintained on HFD for 10 weeks. Regardless of genotype, the body weight of placebo treated OVX mice was increased throughout HFD feeding (closed circle in Figure 3A). Tam treatment resulted in normalized body weight in both genotypes (open circle in Figure 3A). The body weight of E2-treated AF2ERKI females did not normalize and resembled the profile of the placebo treated group (blue open triangle in Figure 3A). The profile of body fat content, detected by DEXA, was parallel to the body weight (Figure 3B). The tissue weight of liver and interscapular fat was not significantly changed by any treatment in either genotype (Figure 3C). In contrast, the tissue weights of visceral and inguinal fats in WT females were reduced by E2-and Tam-treatment; the tissue weight of inguinal and visceral fats in Tam-treated AF2ERKI females was reduced, and E2 treatment had no effect on AF2ERKI tissue weight (Figure 3C). The proportion of inguinal fat content to body weight (relative tissue weight) in the Tam-treated AF2ERKI females was significantly reduced but did not occur in the other tissues (Figure 3D). These results indicate that the lowered inguinal fat accumulation in Tam-treated AF2ERKI females may be a major factor in the lower body fat content, which DEXA detected, in the Tam-treated AF2ERKI mice. Histologically, the fat cells were smaller in the inguinal fat from Tam-treated mice compared to those in the placebo group (Figure 3E). It is likely that the Tam-dependent reduction of inguinal fat mass occurred through prevention of fat accumulation rather than regulation of fat cell number.

The GTT profile showed that the experimental groups with higher body weights (placebo-treated WT and AF2ERKI and E2-treated AF2ERKI) displayed impaired glucose tolerance (Figure 3F). However, regardless of genotype or treatment, the OVX mice responded to insulin when we performed ITT (Figure 3G). Particularly, a significant difference was observed between the HFD-fed intact AF2ERKI and placebo treated OVX AF2ERKI females (blue dotted line and blue line with closed circle respectively; p < 0.0001). Additionally, fasting serum insulin levels in the HFD-fed OVX AF2ERKI females were lower than in the intact AF2ERKI females (Figure 3I) despite no differences in fasting glucose between the HFD-fed intact and OVX AF2ERKI females (Figure 3H). These results suggested that ovarian hormones are involved in the blunted sensitivity to insulin that was observed in HFD-fed intact AF2ERKI females.

E2 treatment induced remarkable uterine growth in the OVX WT females compared to the intact WT (0.186 ± 0.021 mg and 0.091 ± 0.017 mg respectively; p < 0.0001). E2 stimulated hypertrophic uteri in the OVX WT females caused obstructive uropathy in this treatment group. This condition resulted in removal of some mice from the study. On account of this issue, the E2-treated OVX WT group was not included in gene expression analyses.

### 3.4 Differential expression profile of fat metabolism related genes in inguinal and visceral fats

**Figure 4:**
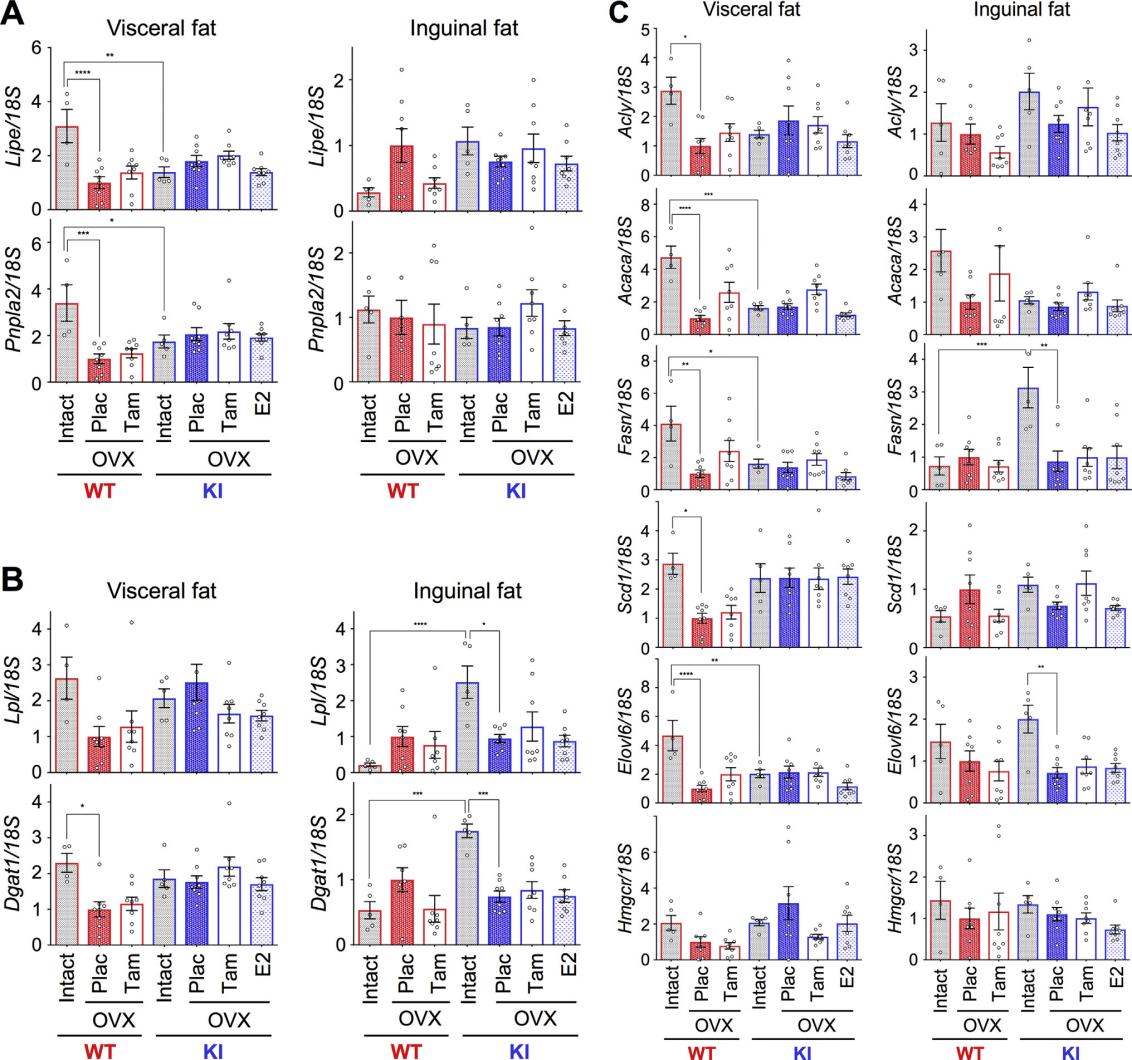
**Differential expression profile of fat metabolism related genes in inguinal and visceral fats.** (**A**) mRNA levels of lipolysis rate limiting enzyme coding genes in visceral fat and inguinal fat. (**B**) mRNA levels of triglyceride synthesis key enzyme coding genes in visceral fat and inguinal fat. (**C**) mRNA levels of fatty acid synthesis and cholesterol synthesis related genes in visceral fat and inguinal fat of HFD-fed intact WT and AF2ERKI (KI) females and in HFD-fed OVX WT and AF2ERKI (KI) females implanted with placebo (Plac), tamoxifen (Tam), or estradiol (E2) pellet for 10 weeks. Data represented as relative mRNA levels compared to the level of placebo treated OVX WT female set as 1. The data shown as mean ± S.E.M.. Two-way ANOVA was performed to indicate significant difference between treatments. *p < 0.05, **p < 0.01, ***p < 0.001, ****p < 0.0001.

**Figure 5:**
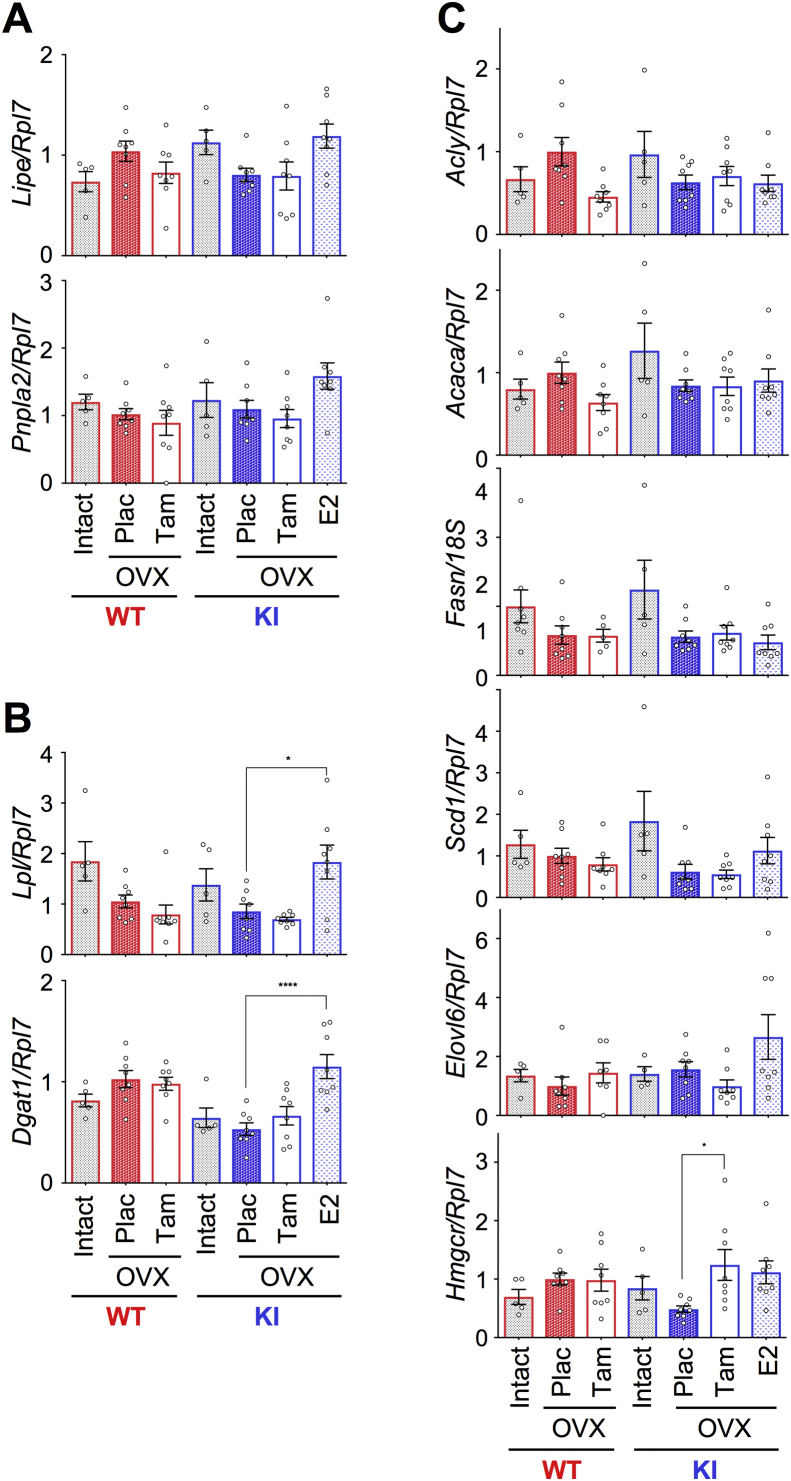
**Expression profile of fat metabolism related genes in liver.** (**A**) mRNA levels of lipolysis rate limiting enzyme coding genes in liver. (**B**) mRNA levels of triglyceride synthesis key enzyme coding genes in liver. (**C**) mRNA levels of fatty acid synthesis and cholesterol synthesis related genes in liver of HFD-fed intact WT and AF2ERKI (KI) females, and HFD-fed OVX WT and AF2ERKI (KI) females implanted with placebo (Plac), tamoxifen (Tam), or estradiol (E2) pellet for 10 weeks. Data represented as relative mRNA levels compared to the level of placebo treated OVX WT female set as 1. The data shown as mean ± S.E.M.. Two-way ANOVA was performed to indicate significant difference between treatments. *p < 0.05, ****p < 0.0001.

To understand the mechanism of AF-1 mediated differential fat accumulation in the adipose tissues, we analyzed the expression profile of lipid metabolism related genes (lipoprotein lipase, *Lpl*; diglyceride acyltransferase, *Dgat1*; hormone sensitive lipase, *Lipe*; adipose triglyceride lipase, *Pnpla2*; ATP citrate lyase, *Acly*; acetyl-CoA carboxylase alpha, *Acaca*; fatty acid synthase, *Fasn*; stearoyl-CoA desaturase 1, *Scd1*; elongation of very long chain fatty acids-like 6, *Elovl6*; 3-hydroxy-3-methylglutaryl-CoA reductase, *Hmgcr*) in inguinal and visceral fats. The expression levels of *Lipe*, *Pnpla2*, *Acaca*, *Fasn*, and *Elovl6* in visceral fat were higher in the intact WT than the OVX WT females. However, such a profile and effect was not observed in WT inguinal fat (Figure 4A,C). In contrast, the expression levels of *Lpl*, *Dgat1*, *Fasn*, and *Elovl6* were higher in the inguinal fat in the intact AF2ERKI than in the OVX AF2ERKI females. Such a effect was not observed in AF2ERKI visceral fat (Figure 4B,C). Differential gene expression profiles in the fat depots observed between intact and OVX animals suggested a differential effect of ovarian hormone(s) on inguinal and visceral fat. The expression of these genes, however, was not regulated by Tam treatment in either of the fat tissues. Additionally, we examined the hepatic expression of these genes and found that the genes were not regulated by Tam treatment except *Hmgcr* (Figure 5). We could not find any correlation between Tam-mediated regulation of fat accumulation and fat metabolism related gene expression. These results led us to hypothesize that the AF-1 mediated preventive effect on fat accumulation may be reflected indirect events such as food intake and/or energy expenditure rather than a direct regulation in adipose tissues.

### 3.5 Activation of ERα AF-1 improved energy expenditure

**Figure 6:**
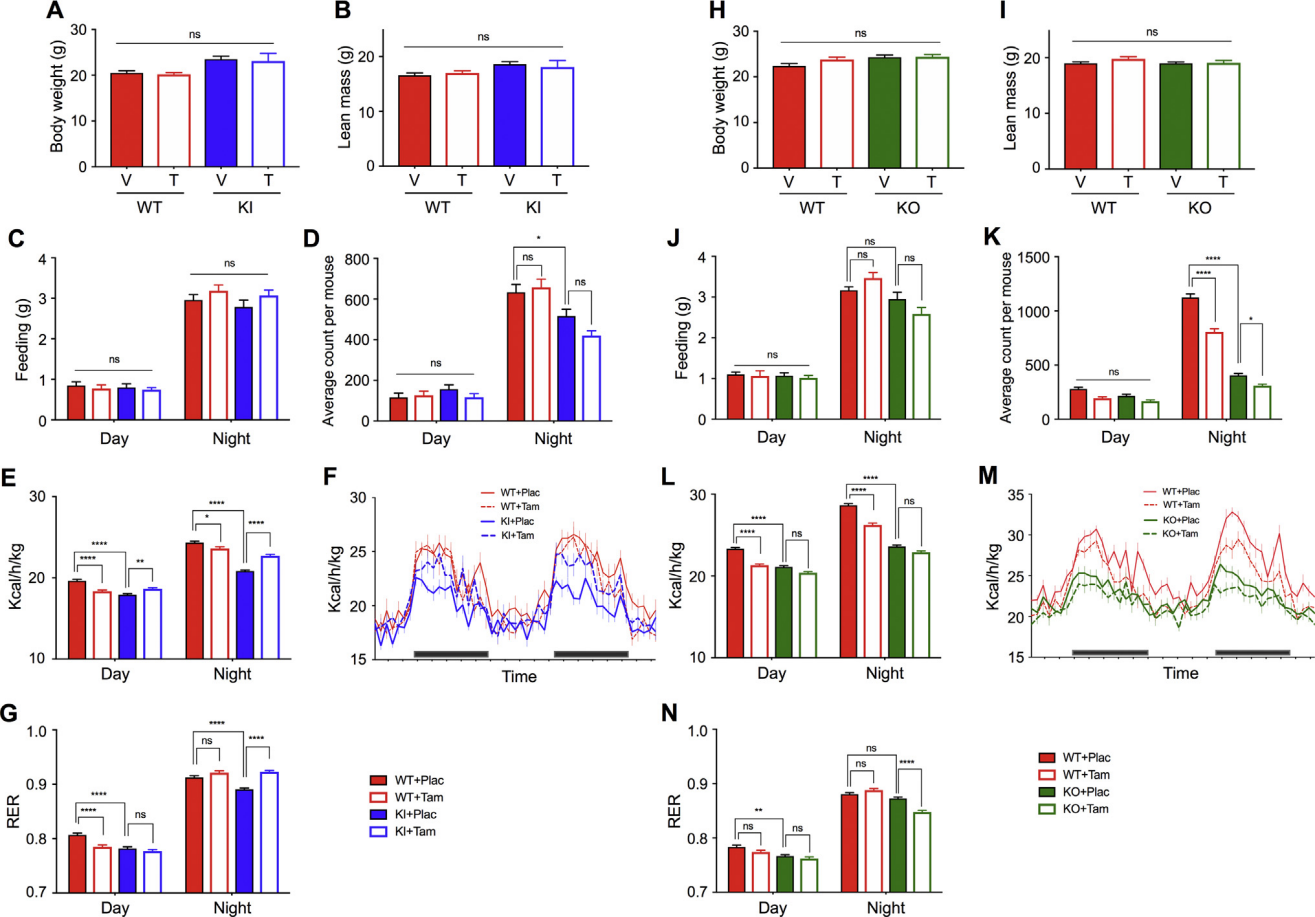
**ERα AF-1 activation improved energy expenditure.** (**A**) Body weight of WT and AF2ERKI (KI) females implanted with placebo (P; WT *n* = 6, KI *n* = 6) or tamoxifen (T; WT *n* = 6, KI *n* = 6) pellet for 14 days. (**B**) Lean mass of WT and KI females. (**C**) Food consumption of WT and KI females in the day (7:00–18:59) and night (19:00–6:59) during the experiment (48 h). (**D**) Ambulatory activity of WT and KI females in the day and night during the experiment. (**E**) Sum of energy expenditure in the day and night periods. (**F**) Energy expenditure at the time. Black bars indicate night period. (**G**) Sum of respiratory exchange ratio (RER) in the day and night during the experiment. (**H**) Body weight of WT and αERKO (KO) females implanted with placebo (P; WT *n* = 6, KO *n* = 6) or tamoxifen (T; WT *n* = 6, KO *n* = 6) pellet for 14 days. (**I**) Lean mass of WT and KO females. (**J**) Food consumption of WT and KO females in the day (7:00–18:59) and night (19:00–6:59) during the experiment (48 h). (**K**) Ambulatory activity of WT and KO females in the day and night during the experiment. (**L**) Sum of energy expenditure in the day and night periods. (**M**) Energy expenditure at the time. Black bars indicate night period. (**N**) Sum of RER in the day and night during the experiment. Data represented as mean ± S.E.M.. Two-way ANOVA was performed to indicate significant difference. *p < 0.05, **p < 0.01, ****p < 0.0001; ns, non-significant difference.

To test this hypothesis, we performed metabolic phenotyping using an indirect calorimetry monitoring system. We used intact animals in this study, because we have observed Tam treatment blocked body weight gain of RD-fed intact AF2ERKI females (data not shown). Mice were single-housed in a metabolic chamber for 72 h to record the levels of oxygen consumption, carbon dioxide production, food intake, and ambulatory activity. To allow for an acclimation period, the data from the final 48 h were used for analysis. Mice with similar body weight and lean-mass were used for this experiment to exclude the parameter of body composition differences for assessing the heat production level (energy expenditure) (Figure 6A,B, H and I). Additionally, we evaluated the energy source of macronutrients (carbohydrate and fat) using the respiratory exchange ratio (RER). Higher RER values suggest higher carbohydrate usage and lower RER values suggest higher fat usage as the energy source (Figure 6G, N). The food intake level in AF2ERKI mice was the same as WT and this was not affected by Tam treatment during the experimental period (Figure 6C). Locomotor activity of the AF2ERKI females at night was significantly lower than in WT females, and Tam treatment did not change the activity in either genotypes (Figure 6D). The energy expenditure of placebo treated AF2ERKI females was significantly lower than that of WT, and that level was restored to the WT level by Tam treatment (Figure 6E, F) with no differences observed in food consumption and locomotor activity. The RER results suggested that the carbohydrate usage for energy source at night was enhanced by Tam treatment to the AF2ERKI females (Figure 6G). To verify whether this event occurred through Tam-mediated ERα AF-1 activation of AF2ERKI females, comparative analysis was performed using the αERKO and WT littermates. The food intake level was the same between genotypes and was not affected by Tam treatment during the analysis (Figure 6J). Locomotor activity of the αERKO females at night was significantly lower than WT females, and Tam treatment did not increase the activity of αERKO female mice (Figure 6K). The energy expenditure in αERKO females was lower than WT and that was coincided with activity (Figure 6L, M). In contrast to the findings with AF2ERKI females, the energy expenditure of αERKO females did not improve with Tam treatment. Additionally, Tam treatment to the αERKO females reduced the carbohydrate usage as an energy source at night rather than enhancement, which was observed in Tam-treated AF2ERKI females (Figure 6N). These results suggested that the Tam-mediated enhancement of energy expenditure in AF2ERKI females was regulated in an ERα AF-1 dependent manner.

## 4 Discussion

### 4.1 ERα AF-1 is involved in metabolic regulation

We have previously reported the generation and use of the AF2ERKI mouse model, which has a mutated *Esr1* genome generating a two amino acid exchange (L543A, L544A) in the AF-2 core domain of the ERα protein coding sequence [11]. This mutation inactivates E2-mediated AF-2 functions, whereas the AF-1 transcriptional activity can uniquely be activated by ERα antagonists, such as Tam and ICI [11], [16], selectively distinguishing AF-1 functionality not only by the loss of function but also experimentally by gain of function. Here we show that AF2ERKI females display disrupted metabolic phenotypes similar to global ERα null mutant mice [2], [15]. Furthermore, Tam treatment prevented HFD-induced obesity in OVX AF2ERKI females, suggesting that ERα AF-1 can control metabolic regulation without AF-2 activation but not the accompanying insulin insensitivity commonly seen in metabolic syndrome.

Recently, Guillaume et al. reported that HFD-induced metabolic disturbances in OVX WT mice were protected by Tam administration [8]. This conclusion is consistent with our findings. Additionally, it was reported that Tam-mediated protection of obesity was not observed in ERaAF-1° mice, which do not have the AF-1 domain. Therefore, the report concluded that selective activation of AF-1 with Tam is sufficient to prevent obesity [8]. Tam is a bifunctional estrogenic compound, which has an AF-1 activation function and an inhibition function against E2-activated AF-2 [6], [7]. The AF-2 activity of ERα should be quiescent under the E2-depleted condition in the OVX females; therefore, the effects of Tam in OVX WT females are likely to arise from AF-1 activation function rather than the inhibition activity of AF-2. This hypothesis is supported by the metabolic phenotype of Tam-treated AF2ERKI female that is a genetically AF-2 quiescent mouse.

The status of ovarian hormones in the ERα mutant mice needs to be considered. Due to the disruption of the estrogen negative feedback regulation, serum E2 and testosterone levels in the intact AF2ERKI females are significantly higher than in WT littermates [11]. This elevated E2 level could affect other estrogen receptors mediated regulation, such as ERβ or G protein-coupled estrogen receptor 1 (GPER) activity. The disrupted metabolic phenotypes of AF2ERKI females suggest that the role of ERβ and GPER in the metabolic regulation may be minor. Moreover, the lack of a preventive effect of E2 treatment to the OVX AF2ERKI mice suggests a minor role of ERβ and GPER in the estrogen dependent regulation of obesity in female mice. Interestingly, the expression of hepatic *Lpl* (lipoprotein lipase) and *Dgat1* (diacylglycerol acetyltransferase), which encodes the key enzymes regulating lipid accumulation, was significantly increased by E2 in the OVX AF2ERKI females (Figure 5B). This might suggest a negative impact of ERβ or GPER on lipid accumulation in the female liver. We did not find any evidence of the positive involvement of ERβ or GPER in the obesity control in this context. Taken together these observations emphasized that the importance of selective activation of ERα AF-1 functionality to control obesity in females.

### 4.2 Differential fat accumulation in inguinal and visceral fats

The DEXA profile showed that the overall fat accumulation of OVX AF2ERKI females was reduced by Tam treatment (Figure 3B). Specifically, the accumulation of inguinal fat was strongly impeded by Tam treatment compared to visceral fat (Figure 3D). The differential characteristics of intra-abdominal (visceral) and subcutaneous (inguinal) fats related to the differential fat accumulation profiles between males and females have been discussed [17], [18], [19]. On average, men have less total body fat but more intra-abdominal adipose tissue, whereas women tend to have more total fat accumulated in subcutaneous depots [19]. The distribution of fat in post-menopausal women shifts to a similar pattern seen in men; however, estrogen replacement therapy prevents intra-abdominal fat accumulation [20], [21], [22]. We found that the genes *Pnpla2* (adipose triglyceride lipase) and *Lipe* (hormone sensitive lipase), which encode the catalytic enzymes for the rate-limiting step of lipolysis, were expressed at lower levels in the placebo-treated OVX WT and intact AF2ERKI visceral fat compared to the intact WT (Figure 4A). The tissue weight of visceral fat in the placebo-treated OVX WT and intact AF2ERKI, compared to intact WT animals, was higher, but this did not reach statistical significance. These results indicate that the reduction of lipolysis in the E2 inactivated females might be a cause of the accumulation of visceral fat. The expression of *Pnpla2* and *Lipe* may be regulated by E2. However, these genes were not regulated in the Tam-treated AF2ERKI females suggesting that the ERα AF-1 is not involved in this regulation, but may be a function mediated by AF-2. Interestingly, such regulation was not observed in the inguinal fat (Figure 4B). E2-depletion caused reduction of lipolysis activity in the visceral fat but not in the inguinal fat which may explain the increase in intra-abdominal fat in post-menopausal women which can be prevented by estrogen treatment.

The genes that encode the key enzymes regulating lipid accumulation (*Lpl* and *Dgat1*) were expressed at higher levels in the intact AF2ERKI compared to the placebo-treated OVX AF2ERKI or intact WT females. This regulation was observed in the AF2ERKI inguinal fat exclusively. Enhancement of lipid accumulation in the inguinal fat may cause preferential fat accumulation of inguinal fat rather than visceral fat in intact AF2ERKI females (Figure 2D). Intact AF2ERKI females have higher serum testosterone levels [11], which need to be considered as this might be a factor in the up-regulation of those genes. We have previously reported that hepatic steatosis in female mice is prevented by estrogen and enhanced by testosterone [15]. The ratio of estrogen and testosterone levels in women is inverted during the menopause period because of the reduction of mature follicles, which express aromatase [23]. The balance of estrogen and testosterone levels is an important parameter to consider in the health of post-menopausal women.

The expression profiles of lipolysis related genes and lipid accumulation related genes were different in the inguinal and visceral fats. These genes were not regulated by Tam treatment in the OVX AF2ERKI tissues even though the fat mass was changed. This result suggests that the ERα AF-1 mediated transactivation in the adipose tissues is not primary regulator of Tam-dependent prevention of fat accumulation. It is likely that the differential responses of the fat accumulation mechanism in the inguinal and visceral fats resulted in a differential Tam effect on AF2ERKI adipose tissues.

### 4.3 ERα AF-1 activation improved energy expenditure

Basal metabolic rate (BMR), occasionally called basal energy expenditure, has been used for standardized measurements of metabolism across different species. The basic requirement for a measure to qualify as basal is that the organism should be at rest, alert, post-absorptive, not growing or reproducing, at a temperature within the thermoneutral zone, and the measurement should be taken during the quiescent phase of its diurnal cycle [24]. Our measurement of metabolic rate in the daytime meets most BMR criteria excluding post-absorptive measures. Thus, we used the term resting metabolic rate (RMR). The RMR of AF2ERKI and αERKO females was significantly lower than the RMR of placebo treated intact WT females. These results suggest that blocking the functionality of ERα AF-2 reduces the RMR of female mice. The lower RMR of AF2ERKI female mice was improved by Tam treatment, which resulted in a similar level as Tam-treated WT females. However, this effect was not observed in the αERKO, suggesting that AF-1 activation improves RMR. Interestingly, the RMR of intact WT females was significantly reduced by Tam-treatment and that level was similar to the Tam-treated AF2ERKI females. It is likely that the RMR regulation through the Tam mediated AF-1 activation function was balanced by AF-2 inhibition activity of Tam in the presence of estrogen in the intact females. We speculate that improved RMR in the Tam-treated AF2ERKI mice reflects a significant induction of energy expenditure during the active period (night) without changing locomotor activity.

There is a long-lasting debate about the link between Tam treatment and body weight gain in breast cancer patients with reports showing contradicting conclusions [25], [26], [27], [28]. Our animal study suggests that the treatment with Tam to the intact WT females reduced the resting-time (daytime) energy expenditure, i.e. BMR. In humans, the reduction of BMR is a possible risk factor for body weight gain. The level of energy expenditure in Tam treated WT female mice during the active period (night) was reflected in the locomotor activity. It is likely that the Tam-reduced BMR could be balanced by the locomotor activity related energy expenditure. Such a consideration has been made as the correlation between weight gain and energy surplus of Tam treated patients [29], [30]. Demark-Wahnefried et al. demonstrated reduced activity with no change in caloric intake in women undergoing chemotherapy who gained weight [31]. Our experimental results support their epidemiological findings. Our results also suggest that estrogen level could affect the balance of Tam mediated ERα AF-1 activation and AF-2 inhibition functions to regulate energy expenditure. The combination of menopause status (hormone levels) and the degree of activity might explain the varying patient reports.

It was reported that the energy expenditure of OVX WT females was lower than that of intact WT females, coinciding with a reduction of locomotor activity and no alteration of food intake [32]. We observed that the locomotor activity of AF2ERKI and αERKO females was also lower than the activity seen in WT females. It is highly likely that the lower locomotor activities reflect lower energy expenditure and that results in the obese phenotype of these genotypes and OVX WT females compared to WT intact females. The differential hormone levels between OVX and intact females need to be considered. Namely, ovariectomy causes reduction of ovarian hormone levels while simultaneously the levels of pituitary and hypothalamic hormones increase in the OVX WT females because of the disruption of negative feedback regulation. Importantly, the profile of pituitary and hypothalamic hormones in AF2ERKI and αERKO females is similar to OVX WT females [1], [11]. This might suggest that the similar phenotypes of locomotor activity observed in the ERα mutants and OVX WT females are caused by the disruption of estrogen negative feedback regulation in the hypothalamus. For instance, in our observations, Tam-treatment did not improve the reduced locomotor activity of AF2ERKI females, and the locomotor activity of intact WT females was not reduced by Tam-treatment as seen in the ERα mutant females. These results may suggest that the locomotor activity in female mice is not regulated by ERα mediated transcription directly. ERβ or GPER might be involved in the regulation of estrogen associated locomotor activity.

Although the locomotor activity of AF2ERKI female mice was not restored by Tam treatment, the level of energy expenditure was restored. Importantly, this effect was not observed in the αERKO. These results indicate that the activation of ERα AF-1 mediated transcription may be involved in controlling the energy expenditure independent from controlling the locomotor activity. The result of respiratory exchange ratio (RER) suggests that Tam-treatment to the AF2ERKI females restored the level of energy (heat) production from carbohydrate metabolism. Further research is needed to determine which organ(s) enhanced carbohydrate metabolism in Tam-treated AF2ERKI females, as that organ might be a direct target of ERα AF-1 mediated regulation.

A previous study reported that weekly food intake was reduced in Tam-treated OVX WT mice but not in αERKO nor ERaAF-1° littermates, concluding that Tam-mediated reduction of food intake causes prevention of body weight gain [8]. On the other hand, our study showed that Tam treatment did not change food consumption during the metabolic chamber experiment. These inconsistent results of Tam-treated mice might have occurred due to the differences of ovarian hormone levels (OVX versus intact) and/or food composition (HFD versus RD). In addition, our analysis period (48 h) might be short to observe differential food consumption. Tam mediated reduction of food intake might have resulted in the Tam mediated enhancement of energy expenditure.

## 5 Conclusions

In summary, the enhancement of energy expenditure in the Tam-treated AF2ERKI females may be a major cause for prevention of fat accumulation. It is likely that the differential fat accumulation mechanisms of inguinal and visceral fats led to differential Tam responses rather than differential ERα AF-1 functionality in those fat tissues. Our report proposes that controlling the ERα AF-1 activity could be a novel target and approach for development of hormone replacement therapies.
